# A simple and fast flow injection amperometry for the determination of methimazole in pharmaceutical preparations using an unmodified boron-doped diamond electrode

**DOI:** 10.5599/admet.1584

**Published:** 2023-01-01

**Authors:** Adison Meoipun, Kantima Kaewjua, Orawon Chailapakul, Weena Siangproh

**Affiliations:** 1 Department of Chemistry, Faculty of Science, Srinakharinwirot University, Sukhumvit 23 Rd. Wattana, Bangkok, 10110, Thailand; 2 Electrochemistry and Optical Spectroscopy Center of Excellence, Department of Chemistry, Faculty of Science, Chulalongkorn University, Pathumwan, Bangkok 10330, Thailand

**Keywords:** Methimazole, boron-doped diamond thin film electrode, flow injection analysis, drug formulations, amperometry

## Abstract

In this work, an automated flow injection analysis (FIA) connected to a boron-doped diamond electrode (BDDE) was originally developed for the analysis of methimazole in pharmaceutical preparations. At a modification-free BDDE, methimazole was easilly oxidized. For the analysis of the mechanisms occurring at the electrode surface, cyclic voltammetry was employed to evaluate the impact of fundamental experimental parameters, such as pH and scan rate, on the BDDE response. For the quantitative detection, the FIA amperometric approach was constructed and used as a fast and sensitive method. The suggested approach provided a broad linear range of 0.5–50 μmol/L and a low detection limit of 10 nmol/L (signal-to-noise ratio = 3). Furthermore, the BDDE was successfully utilized to quantify methimazole in genuine samples from a variety of medicines, and its performance remained steady after more than 50 tests. The findings of amperometric measurements exhibit excellent repeatability, with relative standard deviations of less than 3.9 and 4.7 % for intra-day and inter-day, respectively. The findings indicated that, compared with traditional approaches, the suggested method has the following advantages: quick analysis time, simplicity, highly sensitive output, and no need for complicated operational processes.

## Introduction

Methimazole (2-mercapto-1-methyl imidazole, MMI) is used to treat hyperthyroidism or an overactive thyroid. It is also prescribed before undergoing thyroid surgery or radioactive iodine treatment. The most prevalent cause of hyperthyroidism is Graves’ disease, an autoimmune condition caused by antibodies that bind to receptors on thyroid hormone-producing cells in the thyroid gland, inducing excessive thyroid hormone synthesis. By mixing iodine with a protein called thyroglobulin, an enzyme called peroxidase generates thyroid hormones, such as thyroxine (T4) and triiodothyronine (T3). MMI stops iodine and peroxidase from producing T4 and T3 from their usual interactions with thyroglobulin. Therefore, thyroid hormone synthesis is reduced as a result of this activity. The conversion of T4 to T3 is also hampered by MMI. Because T3 is more effective than T4, it decreases thyroid hormone activity [1,2]. MMI was authorized by the FDA in March 1999 [3]. However, MMI can induce nephritis, liver cirrhosis, skin irritation, allergies, pharyngitis with fever, and other adverse effects [4]. In pharmaceutical, nutrition, and clinical chemistry, accurate drug determination using simple and rapid procedures is a prerequisite.

The analytical methods used for MMI determination are described, including thin-layer chromatography [5], high-performance liquid chromatography with tandem mass spectrometry [6], ultraviolet detection [7,8], spectroscopy [9-11], potentiometry [12], liquid chromatography with amperometric detection [13], and capillary zone electrophoresis [14]. Among these, liquid chromatography is one of the most commonly employed in pharmaceutical research. While it is often utilized in laboratories, there are instances when complicated procedures must be developed and validated. They could also take a lot of time and call for qualified employees. On the other hand, because they are quick, simple, accurate, and exceedingly sensitive, electroanalytical methods have proved effective in detecting a wide range of analytes, including those present in biological and pharmaceutical samples. However, electrochemistry has a limitation in that it is not an automated method causing it not to support routine work. The discovery of a flow-based system led to widespread application in quantitative pharmaceutical analysis. Flow-based technology has been useful and can be an option for routine analysis with a fast response and a substantially lower cost. Moreover, flow injection analysis (FIA) offers the possibility to work in continuous rather than in batch mode, with higher sample throughput and various detectors. Thus, electrochemical techniques have been widely used as a detector in flow injection techniques for pharmaceutical applications [15,16]. One of the most frequent working electrodes used in electrochemical research is the glassy carbon electrode (GCE). Unfortunately, MMI oxidation at the GCE generally has a weak electrochemical response. Therefore, chemically modified electrodes have been frequently documented for the sensitive and selective determination of MMI. Acetylene black/chitosan film [17], MWCNTs [18], MWCNTs/electro-copolymerized cobalt nanoparticle–poly (pivalic acid) composite film [19], and carbon paste electrodes modified with Schiff base complexes of vanadium and cobalt [20,21], nanocomposite of CdS NP–RGO/IL [22], and MWCNT–titanium dioxide nanoparticles [23] are among the modifiers. In several applications mentioned, time was wasted in the modification and pretreatment steps. Furthermore, there is still a significant issue with electrode preparation repeatability.

Based on our expertise, the chemical and physical properties of a boron-doped diamond electrode (BDDE) (low background currents, wide potential window, good resistance to fouling, chemical and mechanical stability, lack of a surface oxide film, and controllable surface termination) have been successfully used in a variety of applications [24-26].

In this regard, BDDE can be realized for the quantification analysis of MMI [27]. However, the difference of this study from the previous work related to the BDDE is that the modification-free or unpretreated BDDE was used to explore the electrochemical property of MMI *via* cyclic voltammetry as a continuation of our previous work on the usage of the BDDE in applications. As previously mentioned, thus, this is the first time that reported the use of an unmodified BDDE as a detector in an FIA system to determine MMI. The online FIA and the promising characteristics of the BDDE enable the detection of extremely low MMI levels. Good analytical detection performances were observed with detection limits in the nanomolar range, strong sensitivity, outstanding response accuracy, and stability. Therefore, the ideal protocol to work with BDDE for FIA enables the electrochemical pretreatment step and sequential detection to simplify regular applications and routine measurements.

## Experimental

### Chemicals and reagents

Without additional purification, all compounds were of analytical grade or above. All solutions and subsequent dilutions were prepared in deionized water. Phosphate solutions (0.1 mol/L, pH 5–8) were made by combining 0.1 mol/L potassium dihydrogen phosphate (Merck, Darmstadt, Germany) and 0.1 mol/L sodium hydrogen phosphate (Fluka, Germany). Phosphate solution (pH 2.5) was prepared from 0.1 mol/L potassium dihydrogen phosphate, and the pH was adjusted with orthophosphoric acid (85 %, Carlo Erba). Phosphate solution (pH 9), 0.1 mol/L, was prepared from 0.1 mol/L potassium dihydrogen phosphate, and the pH was adjusted with 0.1 mol/L sodium hydroxide. Prior to use, the standard methimazole (Sigma-Aldrich, Darmstadt, Germany) solutions were prepared daily using the same solution.

### Electrodes

The equipment utilized for the diamond film generation and the experimental setup have been extensively documented elsewhere [28]. The films were created by microwave plasma-assisted chemical vapor deposition of BDD thin films on highly conductive n-Si (111) substrates. A layer thickness of around 30 mm was attained after 10 hours of deposition. The normal degree of boron doping in the film was around 1021 cm^3^, and the nominal B/C atomic ratio in the gas phase was 1:100. Before usage, the BDDE was cleansed with ultrapure water. For the glassy carbon electrode (GCE, (0.07 cm^2^, Bioanalytical System, Inc.), the pretreatment involved sequential polishing with 1 and 0.05 μm of alumina/water slurries on felt pads, followed by rinsing with ultrapure water prior to use.

### Voltammetric study

An Autolab potentiostat 100 (PG100, Metrohm, Switzerland) with a typical three-electrode setup was used to record the electrochemical signals. The planar working BDDE was pressed against a smooth ground joint at the bottom of the cell, isolated by an O-ring. The geometric area of the working electrode was 0.07 cm^2^. A platinum wire served as the auxiliary electrode, and Ag/AgCl was used as the reference. The placement of the back side of the Si substrate on a brass plate led to an ohmic contact. In the comparative study with the BDDE, a GCE (0.07 cm^2^, Bioanalytical System, Inc.) was also utilized as a working electrode. Cyclic voltammetry was employed to probe the electrochemical reaction. The electrochemical measurements were performed in a Faradaic cage to reduce electronic noise. All experiments were conducted at room temperature.

### Flow injection analysis with amperometric detection

The FIA system included an electrochemical detector (PG100), a peristaltic pump (Ismatec), a thin-layer flow cell (all from Bioanalytical System, Inc.), and an injection port with a 20-μL injection loop (Rheodyne7725). A reagent delivery module regulated the carrier stream, which was 0.1 mol/L phosphate solution (pH 9) at a flow rate of 1 mL/min. To minimize the pulsation caused by the peristaltic pump’s roller alternation, a pulse dampener was used in series. A silicone gasket served as a spacer, and the thin-layer flow cell had an Ag/AgCl reference electrode, a stainless-steel tube as an auxiliary electrode, and an outlet. To decrease electrical noise, the experiments were conducted in a copper Faradaic cage. Before performing the amperometric measurement, a hydrodynamic voltammogram was acquired to obtain the suitable detection potential. After each injection, the peak current and the matching background current were recorded. Hydrodynamic voltammograms were created by plotting this data as a function of applied voltage. The hydrodynamic voltammograms provided a maximum signal-to-background (S/B) ratio since the amperometric measurements were performed at the potential of +0.3 V.

### Sample preparations

Tablets containing MMI were acquired from local pharmacies in Bangkok, Thailand. The medication tablet was homogenized in an agar mortar prior to measuring. The accurate amount of the powdered mass analyte was dissolved in 100 mL of 0.1 mol/L phosphate solution (pH 9) by stirring for 10 min. The macromolecule excipient was separated by filtration with Whatman filter paper (No. 1), and the residue was washed three times with phosphate solution (pH 9). To determine the MMI amount of the tablets, the final test solution was diluted with phosphate solution (pH 9) to keep the MMI concentration within the linear dynamic range (0.5–50 μmol/L).

## Results and discussion

### Voltammetric study

**Figure 1.**
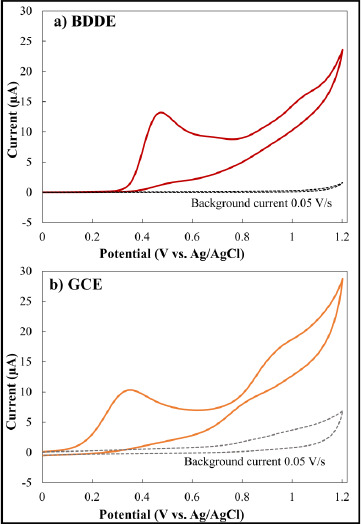
Cyclic voltammograms for a) BDDE and b) GCE versus Ag/AgCl in 1.0 mmol/L MMI in 0.1 mol/L phosphate solution pH 9 (solid lines) and 0.1 mol/L phosphate solution pH 9 (dashed lines). Sweep rate, 0.05 V/s; area of electrode, 0.07 cm^2^

The electrochemical responses of two electrodes (the BDDE and GCE) to MMI were first examined and compared *via* cyclic voltammetry. Figure 1a presents the voltammetric response of the BDDE in a 0.1 mol/L phosphate solution (pH 9) containing 1 mmol/L MMI, whereas Figure 1b shows the voltammetric response of the GCE in the same circumstances. The response at each electrode exhibited no detectable oxidative signals across the investigated voltage range, according to the cyclic voltammograms obtained before the addition of MMI (0.0–1.2 V). Furthermore, the BDDE consistently exhibited lower capacitance than the GCE when background currents at each electrode were evaluated. The response at the BDDE changed when MMI was added to the solution, with a new well-defined electrochemical signal developing at +0.45 V. The MMI response at the GCE, on the other hand, had two pairs of ill-defined peaks observed as an increase in oxidation current at +0.35 V and +0.98 V. For comparison, the CV response of MMI at the BDDE is more significant and has a stronger anodic peak current than bare GCE. This may be concerned with the fact that a BDDE provided a very lower background current than a GCE, suggesting that the BDDE has shown an efficient catalytic activity toward the electrooxidation of MMI. In addition, no additional reductive processes were identified in the voltage range investigated at each electrode when the scan direction was reversed. These irreversible behaviors provide consistent results with a chemically modified electrode reported previously.

### Effect of pH value on the electrochemical behavior of MMI

**Figure 2.**
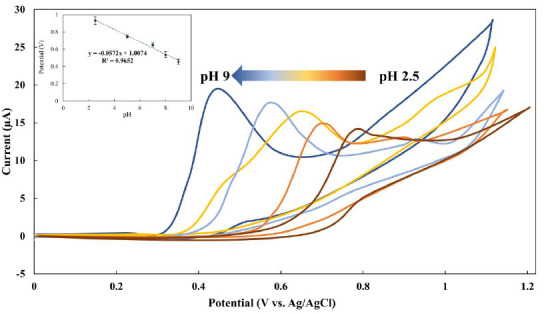
Cyclic voltammogram for 1-mmol/L MMI in 0.1 mol/L phosphate solution at different pH solutions at a BDDE (potential scan rates: 0.05 V/s and area of electrode: 0.07 cm^2^). The calibration curve of the relationship between potential (V) and pH is also presented in the inset of this figure.

Investigating the effect of solution pH on the electrochemical responses of MMI is essential for achieving the best results in high responsiveness. Thus, the electrochemical characteristics of MMI were investigated using a phosphate solution with a concentration of 0.1 mol/L as the supporting electrolyte. The tests were conducted at pH levels of 2.5, 5.0, 7.0, 8.0, and 9.0. According to the findings, MMI may be oxidized in both neutral and alkaline conditions. When the pH value increased, the peak potential shifts toward less positive potentials (Figure 2). Additionally, the oxidation of sulfur-containing compounds at the BDDE can explain this electrooxidation. The oxidation process involved the dissociation of the proton on the thiol group, followed by the electrochemical oxidation of the MMI anion. As a result, MMI easily lost the proton at higher pH levels and formed a more stable reduced form. The use of a phosphate solution at pH 9 yielded the largest oxidation current peak. Hence, this pH was selected as the starting point for all the following studies.

### Scan rate and concentration dependence

**Figure 3.**
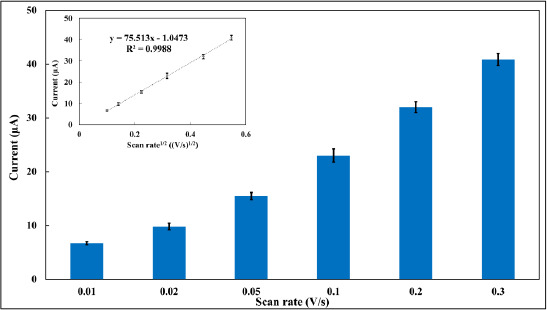
Bar graph for 1 mmol/L MMI in 0.1 mol/L phosphate solution (pH 9) at BDDE for a series of potential scan rate; area of electrode, 0.07 cm^2^. The calibration curve of the relationship between current (μA) and (scan rate)^1/2^ is also presented in the inset of this figure. The measurement obtained based on three replicates (n=3)

To confirm that this oxidative process is diffusion-controlled, an investigation into the effects of scan rate on the electrochemical signal was carried out. As presented in Figure 3, the electrooxidation responses of MMI varied with the scan rate, with the current plateau rising linearly with the square root of the scan rate (R^2^ = 0.9988). Such a relationship indicates that MMI oxidation at a BDDE surface is really controlled by the diffusion process.

**Figure 4.**
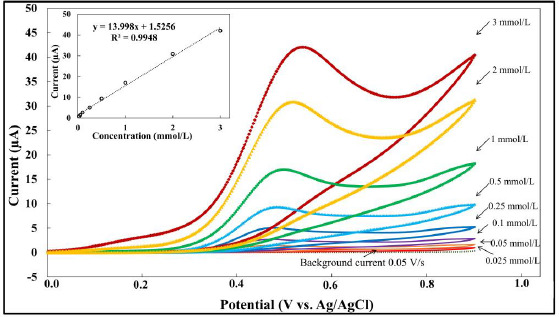
Cyclic voltammogram for MMI in 0.1 mol/L phosphate solution (pH 9) at BDDE for a series of methimazole concentrations. The potential scan rate was 0.05 V/s; the area of electrode, was 0.07cm^2^. The calibration curve is presented in the inset. The measurement obtained based on three replicates (n=3).

In order to perform a preliminary study on the analytical effectiveness of MMI at the BDDE, cyclic voltammetry was used to measure the change in voltammetric current as a function of various MMI concentrations. It was discovered that the response of the BDDE was linear over the concentration range of 0.025-3.0 mmol/L, with a limit of detection of 50 μmol/L at signal-to-background (*S*/*B*) = 3. The data is depicted in Figure 4. However, the obtained linear range and LOD are insufficient for the quantitative detection of MMI in applications such as biological samples or pharmaceutical preparations. Therefore, the use of a highly sensitive electrochemical technique is required.

### Electrochemical behavior of MMI at the BDD electrode

To examine the mechanism of electrooxidation of MMI at the BDDE interface, the electrooxidation behavior of MMI was measured *via* cyclic voltammetry at various potential scan rates. The oxidation current linearly increases with the square root of the scan rate, as presented in Figure 2. In addition, the obtained linear relationship between *I*_pa_ and the *ν*^1/2^ (y = 75.513x −1.0473; *R*^2^ = 0.9988) was used to calculate the diffusion coefficient of MMI (*D*_0_) using Andrieux and Savèant’s theoretical model (Eq. 1):


(1)

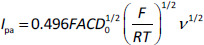



where *F* is Faraday’s constant = 96,485 C/mol, *R* = 8.31447 J/mol·K, *T* = 298 K, *A* = 0.07 cm^2^; *C*_s_ is the MMI concentration = 1 × 10^−6^ mol/cm^3^, *I*p is the oxidative current of MMI, and *ν* is the scan rate. The calculated diffusion coefficient (*D*_0_) of MMI was found to be 1.30 × 10^−5^ cm^2^/s. The Randles–Ševčík relationship for the irreversible process (Eq. 2) was employed to calculate the number of electron transfers at the electrode interface:


(2)

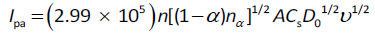




(3)

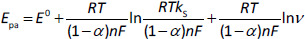



The factor (1-α)*n_α_* was firstly calculated from the linear regression (Eq. 3) between *E*_pa_ and ln *ν* (*y* = 0.0223*x* + + 0.7576; *R*^2^ = 0.9963) and was found to be 1.15. After calculating the important factors, the number of electrons involved in the electrooxidation of MMI was calculated with Eq. 2 and was found to be n = 0.92 (nc1).

Moreover, it was found that the peak potential of MMI shifted to the negative potential by increasing pH. This is predictable due to the participation of proton in the oxidation process of MMI [18,19]. The Nernst equation (Eq. 4) was used to describe the relationship between *E*_pa_ and pH (inset in Figure 2) The linear relationship between the pH values and oxidation peak potentials was plotted (*y* = −0.0572*x* + 1.0074; *R*^2^ = 0.9652).


(4)

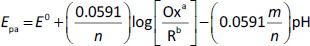



where *m* and *n* are the number of protons and electrons at the electrode interface, respectively. The slope was found to be 57.2 mV, which is in agreement with the theoretical slope (2.303 *mRT*/2*F*) of 59 mV/pH. Based on the calculation, *m* = *n* = 1, since the results show that the quantities of electrons and protons exchanged at the electrode surface are equal. Consequently, the possible mechanism of MMI at the BDDE involves one electron and one proton.

### Hydrodynamic voltammetry

**Figure 5.**
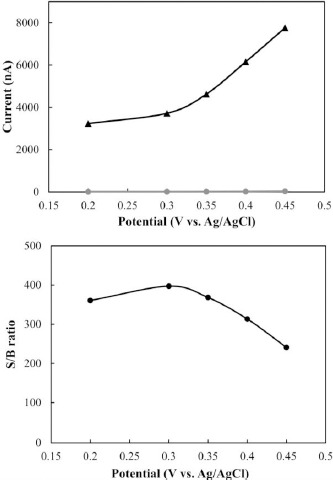
(a) Hydrodynamic voltammogram of (-•-) 0.1mol/L phosphate solution (pH 9, background current) and (-▲-) 100 μmol/L of MMI in 0.1 mol/L phosphate solution (pH 9) with repeated four injections of analysis using 0.1 mol/L phosphate solution (pH 9) as a carrier solution. (b) The hydrodynamic of the signal-to-background (S/B) ratio. The flow rate was 1 mL/min.

Hydrodynamic voltammetry is a helpful technique to figure out what potential to use for FIA/amperemetric detection. A standard solution of MMI was repeatedly injected, while the FIA/amperemetry working potential was adjusted at +0.20, +0.30, +0.35, +0.40 and +0.45 V, respectively. Figure 5a shows the hydrodynamic voltammogram of the standard solution, which contains 100 mol/L of MMI. The current response of MMI increased as the applied voltage increased; however, it did not reach its maximum value. To reach the maximum potential point, the S/B ratio was computed (Figure 5a) at each point to construct a graph as a function of S/B ratios and applied potential (Figure 5b). In this work, +0.30 V was selected as the optimum detection potential for subsequence work owing to high sensitivity and low noise. Remarkably, the reference electrode used for cyclic voltammetry and flow system is different. Thus, it may lead to a little different detection potential being obtained between batch and flow systems.

### FIA coupled with amperometric detection

**Figure 6.**
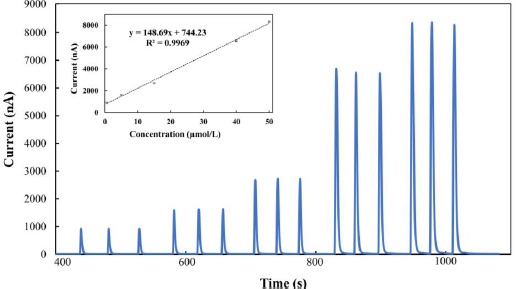
The flow injection analysis results of increasing concentrations of methimazole at a BDDE in 0.1 mmol/L PBS at pH 9. Methimazole concentrations: 0.5, 5, 15, 40, and 50 μmol/L. Inset: A plot of anodic peak currents versus increasing concentration of methimazole.

After setting the suitable detection potential for the FIA technique, amperometric measurements were carried out in the phosphate solution (pH 9) containing various MMI concentrations in order to get the analytical curve. The FIA current-time response for different MMI concentrations (Figure 6) demonstrated a linear relationship between the current values (at +0.30 V) obtained and the MMI concentrations exhibited from 0.5 to 50 μmol/L with a correlation coefficient of 0.9969.

The linearity is disrupted at concentrations greater than 50 μmol/L. The experiment revealed that the detection limit was found to be 10 nmol/L (signal-to-noise (*S*/*N*) ratio = 3). As demonstrated in Table 1, the proposed method gave the lowest detection limit compared to previously published studies, particularly those using chemically modified electrodes. This means that a BDDE may detect MMI with extreme sensitivity while maintaining its speed and ease of use. Furthermore, because it allows for rapid real-time measurements and eliminates the need for electrode cleaning, this system is ideal for pharmaceutical analysis.

### Determination of MMI in commercially available pharmaceuticals

The use of the FIA amperometric approach for the assessment of MMI in commercially available drug samples was performed to test the analytical validity of the suggested method. However, the active ingredients of MMI tablets contain lactose monohydrate, magnesium stearate, starch, corn, and talc [35]. These substances are non-electroactive species that cannot be oxidized at the same oxidation potential as MMI at a BDDE. To ensure that the effects of matrices that may be present in real samples can be reduced, the MMI content was calculated using a standard addition method. The recovery of this method was determined by calculating the percentage of recovery after adding known amounts of standard MMI to the sample solutions. Table 2 presents the recovery for intra-day and inter-day from the determination of MMI using the proposed method. On the basis of intra-assay, the method's precision was established. Three concentrations of the added solution (0.33, 0.65, and 1.31 μg/mL) were selected for the investigation to check the results obtained from the low, medium, and high concentrations with respect to the probable range of interest in the samples. The results obtained from 10 injections were within 3.9 % of the relative standard deviation (RSD, %). The RSD values for the day-to-day assays of MMI were also investigated. It was discovered that during a week in the same laboratory, the %RSD measurements did not vary by more than 4.7 %. These findings indicate that BDDE detection was very reproducible and accurate. In addition, the BDDE were tested for their long-term stability in continuous operation (50 injections). They generated highly repeatable responses from day to day, and the responses were also repeatable after several days in the laboratory environment. The intra-day recovery from the proposed technique was between 95.2 and 101.7 %, whereas the inter-day recovery of MMI was between 93.5 and 103.8 %.

As presented in Table 3, for all the five supplements tested, the experimentally determined values were quite close to the manufacturer’s claims. These results were compared with those obtained using the HPLC-UV technique, and statistical analysis was conducted using a paired *t*-test with a 95 % confidence interval. The null hypothesis was supported by the paired two-tailed test, which yielded a computed *t-value* of 0.906, less than the crucial *t*-value (2.776). A summary of the findings is presented in Table 3, the results of the FIA combined with BDDE amperometry are comparable and not significantly different from those obtained using the traditional HPLC-UV method.

## Conclusions

A simple and fast electrochemical method for the determination of MMI was successfully developed using an automatic FIA connected to an unmodified BDDE. The results indicate that BDDE is a promising material for further research in terms of repeatability, sensitivity, and low background current. Among other interesting features of the approach, it was described as rapid, simple, precise, and extremely sensitive, making it useful for the MMI analysis and quality control of pharmaceutical products. Furthermore, this method may be successfully used to determine MMI in commercially available medications, with results that match the label and standard procedure. This evidence will help understand specific drug pathways and metabolism, leading to their development in the various phases of pharmaceutical research as well as clinical investigations of the MMI levels.

## Figures and Tables

**Table 1.** Comparison of analytical performance for the determination of MMI using different analytical methods

Techniques and detectors	Linearity range, mol/L	Detection limit, mol/L	Ref.
**Flow-Injection:** chemiluminescence	1.75110^-5^ – 8.75 10^-4^	8.75810^-6^	[29]
**Capillary Electrophoresis:** a carbon fiber micro disk electrode	10^-7^ – 2.0 10^-4^	5.00510^-8^	[30]
**Amperometry:** Screen-printed enzymatic biosensor modified with carbon nanotube	7.4710^-8^ – 6.35 10^-5^	5.60510^-8^	[31]
**Differential pulse voltammetry:** Carbon-paste electrode modified with a chiff base complex of cobalt	10^-6^ – 1.0 10^-4^	5.00510^-7^	[21]
**HPLC:** Spectrophotometer detector	5.0510^-8^ – 5.5 10^-7^	7.40710^-5^	[32]
**Differential pulse voltammetry:** acetylene black/chitosan/GCE	10^-7^ – 2.0 10^-5^	2.00210^-8^	[17]
**Square wave voltammetry:** Fluorine-doped tin oxide electrodes	6.0610^-6^ – 240 10^-6^	1.98110^-6^	[33]
**Square wave voltammetry:** GCE	7710^-6^ – 1.3 10^-5^	3.70310^-6^	[34]
**Square wave voltammetry:** Pretreated anodically BDDE	4.38×10^−6^– 2.19×10^−4^	7.45710^−7^	[27]
**Amperometry:** Flow-injection analysis/unmodified BDDE	0.5010^-6^ – 50 10^-6^	10^−8^	This work

**Table 2.** The intra- and inter-precisions and recoveries of the FIA-BDDE method (n = 10)

Samples	Spiked level, Sg/mL	Intra-day		Inter-day	
		Mean of recovery, % (± SD)	RSD, %	Mean of recovery, % (± SD)	RSD, %
Sample 1	0.33	95.3 ± 1.5	3.9	93.5 ± 2.1	4.5
	0.65	96.5 ± 2.6	3.4	97.4 ± 2.3	3.6
	1.31	101.4 ± 1.1	2.6	100.8 ± 1.8	2.8
Sample 2	0.33	98.4 ± 1.2	3.8	99.2 ± 0.7	4.7
	0.65	99.1 ± 1.6	2.6	101.2 ± 2.2	3.1
	1.31	101.2 ± 1.2	1.5	100.7 ± 1.8	2
Sample 3	0.33	96.4 ± 2.2	3.8	93.5 ± 2.4	4.1
	0.65	101.6 ± 1.9	2.6	103.7 ± 2.2	3.1
	1.31	98.8 ± 1.4	1.6	100.1 ± 1.5	1.9
Sample 4	0.33	95.4 ± 3.3	3.5	94.8 ± 2.7	3.9
	0.65	101.2 ± 2.1	2.1	103.8 ± 1.9	2.9
	1.31	99.3 ± 1.3	2.5	102.3 ± 1.7	2.2
Sample 5	0.33	95.2 ± 2.5	3.3	93.5 ± 1.8	4.4
	0.65	100.8 ± 2.0	2.9	99.8 ± 1.9	2.7
	1.31	101.7 ± 1.4	1.2	103.8 ± 1.2	1.3

**Table 3.** Determination of the MMI levels in different drug samples (n = 5) using the traditional HPLC-UV method and the developed FIA-BDDE method reported herein.

Sample	Amount drug label, mg/tablet	Amount found, mg/capsule (± SD)		Recovery, %	
		FIA-BDDE ^a^	HPLC-UV ^b^	FIA-BDDE ^a^	HPLC-UV ^b^
Sample 1	5	4.93 ± 0.2	4.88 ± 0.5	98.6 ± 1.3	97.6 ± 1.5
Sample 2	5	4.90 ± 0.8	4.93 ± 1.7	98.0 ± 1.7	98.6 ± 1.3
Sample 3	5	4.99 ± 1.7	5.22 ± 1.5	99.8 ± 3.5	104.4 ± 2.3
Sample 4	5	4.96 ± 2.5	4.95 ± 2.4	99.2 ± 2.4	99.0 ± 1.9
Sample 5	5	5.03 ± 1.4	5.05 ± 1.3	100.6 ± 2.7	101.0 ± 2.3

a = This developed method

b = Traditional method
